# Reflection of illness and strategies for handling advanced lung cancer – a qualitative analysis in patients and their relatives

**DOI:** 10.1186/s12913-017-2110-x

**Published:** 2017-03-02

**Authors:** Anika Sparla, Sebastian Flach-Vorgang, Matthias Villalobos, Katja Krug, Martina Kamradt, Kadiatou Coulibaly, Joachim Szecsenyi, Michael Thomas, Sinikka Gusset-Bährer, Dominik Ose

**Affiliations:** 1 Department of General Practice and Health Services Research, University Hospital Heidelberg, Im Neuenheimer Feld 130, 69120 Heidelberg, Germany; 2 Internistische Onkologie der Thoraxtumoren, Thoraxklinik im Universitätsklinikum Heidelberg, Translational Lung Research Center Heidelberg (TLRC-H), Member of the German Center for Lung Research, Heidelberg, Germany; 3 Department of Population Health Sciences, Health System Innovation and Research, University of Utah, Salt Lake City, UT USA

**Keywords:** Lung neoplasms, Palliative care, Health services research, Qualitative research

## Abstract

**Background:**

Lung cancer patients are often diagnosed in an advanced stage of disease. In a situation of palliative treatment, both patients and their relatives experience existential burden. Evidence suggests that multi-professional teams should deal with them as dyads. However, little is known about differences in their individual situation. The purpose of this study is to explore and compare reflections that arise out of the context of diagnosis and to compare how patients and their relatives try to handle advanced lung cancer.

**Methods:**

Data was collected by qualitative interviews. A total of 18 participants, 9 patients diagnosed with advanced lung cancer (ICD- 10 C-34, stage IV) starting or receiving palliative treatment and 9 relatives were interviewed. Data was interpreted using qualitative content analysis.

**Results:**

Reflection aspects were “thoughts about the cause”, “meaning of belief” and “experience of inequity”. Patients often experienced the diagnosis as inequity and were more receptive for believing in treatment success. The main strategies found were “repression”, “positive attitude”, “strong focus on the present” and “adjustment of life terms”. Patient and relative dyads used the same strategies, but with different emphasis. That life time is limited was more frequently realized by relatives than by patients.

**Conclusion:**

While strategies used by relatives are similar to those of patients’, they are less reflective and more pragmatic in terms of handling daily life and organizing care. The interviewed patients were mostly not able to takeover these tasks. To strong was their belief in treatment success, their repression of the future and the focus on the present. This implicates, that in terms of end-of-life care, relatives are important to reach patients who are often not receptive to this topic.

**Electronic supplementary material:**

The online version of this article (doi:10.1186/s12913-017-2110-x) contains supplementary material, which is available to authorized users.

## Background

Lung cancer is one of the most common cancers in both men and women in the world and at the same time the most common cause of cancer death [1, 2]. In the US and in Europe it has the poorest five-year survival rate among all cancer types of approximately 15% [3, 4].

Within the lung cancer trajectory, symptoms often become severe and have been linked to high physical and psychological burden and distress [5]. In comparison to other cancer sites, lung cancer patients declare the highest rate of distress [6, 7]. Additionally, lung cancer is predominantly diagnosed in an advanced state of the disease [8, 9] which means that the prognosis is less than one year in median [10] and palliative care constitutes a fundamental part of the treatment approach. Recent studies about the early integration of palliative care in the trajectory of cancer, found a better quality of life (QOL) and even a benefit in overall survival [11, 12].

Because in palliative care patients and caregivers represent an inseparable ‘unit of care’, these caregivers (often relatives) need to be supported as well [13, 14]. High levels of distress in family caregivers of patients with advanced lung cancer are unfortunately very common [15]. Reasons for this are e.g., facing an uncertain future, dealing with the patient’s emotions, and managing day-to-day tasks [16]. Often, patients and relatives find themselves in a situation confronted with poor prognosis, where facing death was a prime concern [17]. Depression and anxiety scores are high in couples were one partner is affected by advanced lung cancer [18].

However, little research is done to evaluate how both patients and relatives, in a situation of advanced disease and palliative treatment, reflect their illness and try to handle it [19–23]. Even less research is done to compare their situations [17, 19, 21].

This analysis is focused on the reflection of illness and strategies to handle advanced lung cancer of both patients and relatives, comparing their individual situations. We dissociate from the approach of Lazarus and Folkman, who understand coping as a mechanism to regulate emotions as well as to solve problems [24], rather using a health services research hands-on approach than a psychological approach [25, 26]. This represents another perspective via which challenges in health care may be identified in particular.

## Methods

### Study design

The ‘Thoraxklinik’ (Hospital for Thoracic Diseases at the University of Heidelberg), is one of the largest lung cancer centers in Germany, receiving over 1000 newly diagnosed patients each year, 50% in metastatic stage and thus in need of palliative care. A qualitative study named ‘Aims of health care and individual quality of life for patients with limited prognosis’ was started in cooperation with the Department of General Practice and Health Services Research (University Hospital Heidelberg) to improve palliative care within the trajectory of metastatic lung cancer.

A qualitative, exploratory study design was chosen to allow intensive exploration of individual experiences and needs of both patients and relatives, in comparison. The following general research question was explored within this analysis: ‘How do patients and their relatives reflect the disease and which strategies do they use to handle advanced lung cancer?

Ethical approval was given by the Ethics Committee of the University Hospital Heidelberg (S-589/2014). All participants gave their written informed consent and their anonymity and confidentiality was ensured throughout the study.

### Study sample

All patients with ICD-10 diagnosis C-34, stage IV and their relatives were potential participants in this study. Further eligibility criteria were 18 years of age or older, absence of acute psychiatric illness as well as of middle to severe form of dementia and constricted ability to communicate. Patients were recruited from the Thoraxklinik’s patient population. Almost all patients were receiving palliative tumor-specific therapy depending on their individual moment within the trajectory of disease. They were asked for their interest in study participation by an oncologist. As they were often accompanied by relatives, relatives could be asked simultaneously. Potential participants were given detailed information about the study before consenting.

### Data collection

The basis for conducting interviews with patients and relatives were semi-structured, pilot-tested interview guides Additional file 1. Topics and questions of these interview guides are based on theoretical considerations, expert discussions and an extensive literature review. There is the possibility to ask open questions allowing participants to explore new arising issues so that the principle of both to be theory-driven and openness of qualitative research is taken into account. It was planned to conduct at most 32 interviews, respectively 16 with patients and 16 with relatives, but only if saturation of data was not reached before. The duration of the interviews ranged from 30 to 90 min. The interviews were performed with patients and relatives separately to minimize response bias and were conducted by experienced scientists and doctoral candidates from the Department of General Practice and Health Services Research in a private room in the hospital. All interviews were audio-taped and transcribed verbatim.

### Data analysis

The transcribed texts of patients’ and relatives’ interviews, including pseudonymisation, were the basis of qualitative content analysis, using the software ATLAS.ti (version 7.5). A search grid (category system) was formed, on the one hand based on the interview guides, and on the other hand adapted during the process if the data showed new or additional information that did not fit into the category system yet. This represents the approach of both deductive and inductive development and application of categories. Step by step while analyzing the first interviews, the researcher team (two doctoral candidates in medicine, one health services researcher and one psychologist) developed a system of categories and subcategories that were clearly defined and linked with representative examples from the original texts. Within our multi-professional researchers team all aspects were discussed and further modified until a consensus of the final category system was achieved. We analyzed interviews until saturation of data was reached, i.e., there was no additional information gained.

## Results

From March until July 2015, 18 interviews with a total of 9 patients and 9 relatives were conducted. The patients’ age ranged between 55 and 79 years (median 63 years) and the relatives’ age ranged between 51 and 66 years (median 54 years) (Table 1).

**Table 1:** Demographics and participants characteristics

	Patients	Relatives
N	9	9
Sex (male)	66.6% (*N* = 6)	33.3% (*N* = 3)
Age (years)^a^	63 (55;79)	54 (51;66)
Family background (spouses)	-	66.6% (*N* = 6)

^a^ *M* median

### Reflection of illness

We identified themes from the data from patients and relatives and built up three main categories. These categories were “thoughts about the cause”, “meaning of belief” and “experience of inequity”. Comparing patients and relatives, there were more aspects respectively on the part of the patients. We describe each category below (Table 2).

**Table 2:** Reflection of illness

Category	Sub-category	mentioned by
Thoughts about the reason	Why-me-question	a, b
	Cancer causing factors	a, b
	Influence of God	a, b
	Struggle with one-self and others	a
	Mischance, hazard	a
	Finding no sense	b
Meaning of belief	Praying as a resource	a, b
	God-given future	a
	Illness testing the belief	b
	Belief as a source of strenght	a
	Experience of injustice	a
	Belief in therapy	a
Experience of inequity	Cancer despite no smoking	a, b
	Cancer despite healthy life	a
	Illness unjust	a
	Trajectory of illness unjust	a

a = patients; b = relatives

### Thoughts about the reason

Patients emphasized that they asked themselves ‘Why me’, to get an answer concerning cancer. It can be distinguished between patients who repeated questioning and patients who refused to think about it again and again. While some patients could not find an answer concerning ‘why me’, others thought about cancer-causing factors. These thoughts were often related to nicotine, rarely to asbestos, and stress was also considered. Thinking about smoking, patients struggled with themselves. They wondered, if it was their fault and if they could have prevented cancer by quitting smoking earlier or by never having started it. They struggled with others, who presented smoking as a normal activity but were a bad example in hindsight.

*‘Because grandfather smoked, too, I meant no harm by it.’ [PAT 2: 163]*

Another perspective was that work and leisure time led to stress because of the working atmosphere and a lack of time to rest at home. However, there were more ideas, how patients tried to find an explanation for cancer. On the one hand, cancer was understood as a punishment of God. On the other hand, cancer was explained as hazard, like an accident by car.

*‘[…] One gets it, the other does not, one is knocked down, the other falls down a tree.’ [PAT 5: 726]*

Relatives also asked themselves ‘why me’, not focusing on their own situation, but on the patient’s. They spoke about it with the patient and other family members, but found this topic difficult. While some relatives could not find a sense, others thought about cancer-causing factors, similar to patients. They considered smoking more carefully, underlining that it could be a cause, but not for sure.

*‚He has been a smoker, right, and for sure: if you have this type of cancer, you say: ok… may be, yes… maybe, but does not have to be always.’ [REL 1: 57]*

It became apparent that relatives also considered stress, e.g. explaining that patients did not allow themselves rest in their leisure time, because of building the house on their own. Additionally, relatives tried to find an explanation for cancer with other ideas. Similar to the patient’s perspective that it was a punishment by God, there was the perspective of relatives that it was a trial by God: If God did not want cancer to be there, the patients would not be ill.

### Meaning of belief

All in all, religion was brought up only sporadically. Praying helped patients to communicate their emotions and thoughts. But sometimes patients were not sure if their prayers would find a destination. The Christian belief in general meant a source of power. One important statement contained the idea that the future was God-given. Patients related this idea to the response to the treatment, having no influence on it.

It became apparent, that patients believed in chemotherapy. There was a positive attitude towards therapies and their potential to control or maybe downsize the cancer. Patients who thought they lived devotionally experienced desperation and injustice because they felt punished in spite of that.

*‘May one be Christian and think what he or she wants to think, I find it unjust.’ [PAT 2: 135]*

Relatives sometimes doubted the influence of belief and prayers concerning the illness trajectory. They accepted other family members’ efforts to pray for the patient, but did not believe in it for themselves. Related to the perspective that cancer had to be a trial by God. His support was put into question.

*‘I really just have to say- is he [God] able to help us? […] because when I think about it – what we have gone through … has he intended it like that? […] If he had wanted to help us, he would not have tolerated it? […] Is this an examination? - If he did not want it, it would not happen?’ [REL 3: 491–497]*

### Experience of inequity

It was evident that patients found their illness unjust at different points. Cancer in general seemed unjust, as one patient talked about a neighbor dying from cancer who had an exposition to asbestos in his job as a firefighter. The patient underlined the neighbors’ own innocence in this situation. Though patients considered smoking as cause of cancer, they experienced inequity in the situation of having quitted smoking before diagnosis.

*‘[…] it was – really weird. I smoked. Smoked for 30 years. Not much […] and in February I stopped. And in May, I get the diagnosis: Lung carcinoma. There I thought: this pulls the rug out from under someone.’ [PAT. 8: 17]*

Other patients have never smoked their whole life and experienced inequity, too. Besides no leading smoking, a healthy way of life, (e.g. little alcohol) led patients to the experience of inequity. To a lesser degree, the trajectory of the illness was responsible for the feeling of inequity. As one patient regarded his friends’ outcome, living years later after diagnosis of lung cancer, he could not recognize why he should have such a poor prognosis.

*‘So I fail to see it, I know colleagues in the clinic, who went through similar situation 15 years ago, who are clean for 7 years now, so why should not I succeed as well?’ [PAT 8: 121]*

Relatives experienced injustice for their partners, less for themselves. The importance of not smoking or having quitted smoking represents the main aspect, although a healthy way of life was mentioned, too.

### Strategies for handling cancer

We identified four main strategies how patients and relatives handle cancer. These strategies were “repression”, “positive attitude”, “strong focus on the present” and “adjustment of life terms”. Both patients and relatives had a similar amount of aspects. We describe each strategy below (Table 3).

**Table 3:** Strategies for handling cancer

Category	Sub-category	mentioned by
Repression	Repress thoughts of cancer	a, b
	No talking about deterioration and death	a, b
	Cope on my own	a, b
	Distraction in general, avoidance of social media	a, b
Positive attitude	In general, towards therapy	a, b
	Comparison: other patients more distressed	a, b
	Hold on to plans and aims for the future	a, b
	Openness of handling the illness	a, b
	Cancer as a challenge	b
	Irony and humor	b
Strong focus on the present	Live from one day to the other	a, b
	Live as usual	a, b
	Do the best of life	a
	Relish every left day	b
	No changes	a
	Switch off, be active	b
Adjustment of life terms	Hurry less, relax	a, b
	Talking to cancer, take over control	a, b
	New tasks: more praying, documentation	a, b

a = patients; b = relatives

### Repression

Repression and avoidance represented important characteristics in our analysis. While many patients repressed thoughts about cancer in general, they admitted that they refused to talk about deterioration and death as well. They wanted to broach the issue of becoming in need of care or dying later, at an indefinite point.

One patient recognized that within his family, people avoided talking about cancer and death as they tried to convince the patient not to think too negatively. Another patient avoided talking about cancer to his mother as he suspected her negative emotions and their expression. Distraction was commonly used to inhibit negative thoughts.

The fact that few patients tried to cope on their own represented another aspect of repression: The importance of the authority of soul became apparent.

*‘When it comes to my soul and how I get along with my soul, I want to handle on my own and until now I, guess so, have succeeded it.’ [PAT 1: 617]*

Relatives used repression and avoidance as well. They refused to talk about deterioration and death since they thought it would be a burden for their partners. They also wanted to protect patients from negative thoughts and saw no need to talk about it in a situation of physical wellbeing. Trying to find activities for patient’s distraction represented another aspect of repression and avoidance.

*‘We kept him [husband] busy for some time, for eight days, [Name of a family] they got a new living room and so we coated a little and he installed the parquet floor together with his grandchildren. […] Saturday we sent him into the garden […].‘[REL 3: 563–579]*

Further, it came to avoidance of social media to stay emotionally strong, in a situation of high vulnerability in the last episode of life. Although almost all relatives emphasized, that they wanted to communicate with family and friends, some were not ready to inform others, e.g. their neighbors, because they needed to realize and cope on their own first.

*‘They all would be there at any time if I said something, night and day, no problem. But I have to keep them at bay for now.’ [REL 8: 242]*

### Positive attitude

Similar to repression, positive attitude represented another important strategy. Almost all patients used a positive attitude to handle cancer in general and the influence of chemotherapy and psychological burden. This was often used parallel to repression, too. Positive attitude was shown at the beginning of treatment and was often kept up by illness trajectory, e.g. remission or disease stability. This was linked to experience of hope, too.

*‘Looks as if - chemotherapy responds well, as I’m not in pain anymore, certainly due to the analgesics I receive and so the other, because the values of my blood improve - everything is improving.‘[PAT 1: 809]*

Sometimes, patients compared themselves with other patients and assessed themselves more stable, which showed their need for reassurance. Positive attitude was supported by holding on to plans and aims for the future. Return to work meant much besides developing a list with things patients wanted to do or reach in the future. Others had plans to go on holidays and were positive to realize it, too.

We found another aspect of positive attitude in openness. Many patients emphasized that they communicated all information, e.g. concerning illness trajectory, to their family, especially their partner, but also to friends and colleagues. An open and forthright way of communication meant much to patients as they were convinced that hiding thoughts especially towards their partners would be an additional burden.

Openness was further interpreted as an offensive way to handle cancer. It became apparent that sometimes this was the only reasonable way at all.

*‚I’m able to talk about it with everyone and for me it’s the only possibility to handle this kind of illness in a reasonable way, be pro-active.’ [PAT 8: 217]*

Almost all relatives used a positive attitude as well. They considered desperation as futile and realized that they needed to support the patient in his situation, doing one by one. Some were positive about the organization of care in the future, certain to find a solution then. Sometimes, relatives compared their patients’ and their own situation to others’ situation, assessing their own situation more livable, similar to some patients’ approach.

*‘We’re not alone, right? […] When I see other patients, I think perhaps we’re still good, we’re still in line, yes’ - […] where we are able [to bear].’ [REL 3: 117]*

Some relatives saw that patients held on for plans and aims for the future, e.g. financial independence and wondered if these were typical reactions at the end of life. In contrast to the patients, they rarely planned for the future.

Relatives generally underlined the importance of openness towards family, friends and colleagues. Often, they needed communication to stay positive, too. Irony and humor signified another way to preserve positive attitude. Sometimes relatives did not even fear to show their emotions in face of the patients, thinking that sadness was better shared with each other. Rarely, cancer was named a challenge to be fought, together with the patient.

*‘Where we said ok, now we know. Shit. We both are fighters. We’re not used to call ‚please help‘. Further we keep saying ok. Then let’s do the fight.’ [REL 8: 130]*

### Strong focus on the present

A strong focus on the present described a third important aspect of handling cancer. Patients described a life living one day to the other not sure if the next follow-up would make it better or even worse. Often, they were very insecure about the trajectory of the illness and rarely wanted to know how long they will live, so they chose to live as usual.

This also made it possible to do the best of life, as many patients were able to live at home profiting from a good symptom management and out-patient chemotherapy. Few said they had not changed anything in their life at all refusing the understanding of their prognosis completely.

*’Do you have changed something in your life by reason of illness? Nah.‘[PAT 3: 363–365]*

Relatives also focused on the present. They knew that the trajectory of the illness was variable and supported the patients in their short-term planning, emphasizing the importance of going step by step. However, that life time is limited was more frequently realized by relatives than by patients. Relatives tried to do everything for the patient’s quality of life, relishing every day left. They lived as usual as on the one hand, they had to work and manage the household and on the other hand they supported the patients in their need for a normal everyday life.

Sometimes relatives emphasized they needed to switch off, e.g. when thoughts about cancer became too present. Then, they were active, went for a walk or did sports which rather meant normality.

*‘So I seek to do some sports at least once a week. So I do spine gymnastics and train a little at the treadmill, usually I go by train, then I walk from home to the station and back from work and back home, too, so I make about 45 minutes by foot.’ [REL 6: 129]*

### Adjustment of life terms

The most important aspect in ‘adjustment of life terms’ represents rest, to relax. Patients saw their own need to calm down, especially after having suffered from chemotherapy and side effects. This might be expressed in relishing the view from over the veranda and not answering the phone while relaxing. Others hurried less, because they experienced their physical and psychological weakness. Few patients talked to their cancer, trying to take control over it. Other tasks identified were more praying or documentation of illness trajectory.

*‘Well I have, I’ve been praying every evening, just for me, also prior to that, and now, facing difficult situations I do it twice a day sometimes.’ [PAT 4: 533]*

Relatives underlined the patients’ need for rest. They acted like a team as good as possible to calm down together with the patient. They also saw their own need to calm down facing the distress as patients suffer next to them. Talking to cancer could be adopted, realizing the need to support the patient in taking control over cancer. A new task identified was to undertake care for their family, e.g. when this has been the patient’s role before.

*‘[…] to somehow unburden her [mother], to show her:,See, you do not have to manage it, me as your son I do, I take care for my brother’.’ [PAT 5: 11]*

## Discussion

As disease-modifying treatment options for most patients with advanced lung cancer are still missing, research in the last years has also focused on the effects of palliative care. The growing evidence of the benefits for quality of life and even survival have led to a provisional clinical opinion (PCO) of the American Society of Clinical Oncology (ASCO) in 2012 [27] that recommends the integration of palliative care since the moment of diagnosis of metastatic lung cancer. Besides, relevance of Health Services Research has increased as well, due to growing interest in reevaluation and improving of the network for providing health care [26]. Understanding the individual situation and the process patients and their family caregivers are involved in during the trajectory of disease is of fundamental importance to adapt the necessary health services.

The purpose of this qualitative study was to explore reflection and strategies of patients with limited prognosis and their relatives. Our study allowed an intensive and individual examination from the participants’ view. For this paper, we focused on two topics, which were regularly brought up by patients as well as by relatives: Reflection of their illness and strategies for handling their current situation.

In general, patients seemed to struggle with topics concerning reflection intensively and handled their situation in several, variable ways. Relatives brought up reflection less often, but were trying to handle their situation in a similar way to their related patient. They focused on the patients even less than on themselves. Repression together with holding a positive attitude could be identified as the most important aspects.

A multiplicity of coping strategies in patients with lung cancer and their family caregivers have been already described in prior research. The results are of both quantitative and qualitative character and are interpreted in several ways. On the one hand, coping is discussed in the context of psycho-oncology. There, it is often divided into problem- and emotional-focused strategies [19, 28, 29]. We decided against this division, as our study was an exploration through the patients’ and the relatives’ perspective rather than through a psychologic perspective. On the other hand there are also many aspects of coping mentioned within other contexts, as ‘perspectives’ [22], ‘needs’ [17] and ‘creation of hope’ [20].

Overall, our results converge with prior research on how patients and relatives handle advanced lung cancer. An important aspect is represented by trying to maintain a normal life, as mentioned in many studies before [17, 19, 21]. This aspect is covered in our category ‘strong focus on the present’ as ‘live as usual’. Further, repression and avoidance were often emphasized [23, 30] and we identified these strategies as well. According to the fact that patients and relatives often use similar strategies [15, 31] and should be dealt with as dyads [19], it could be interesting to evaluate if and how they influence the patient’s strategy.

What our study adds is the in-depth exploration of reflection in patients and their relatives. Few aspects have been found, e.g. the ‘Why-us’-question in relatives [15], triggered by witnessing the suffering of patients and the feeling of guilt [22] in patients themselves. We found that especially the patients experience inequity from the point of diagnosis onwards and that they wonder about a possible cause of their illness in detail.

Patients sometimes develop a belief in therapy and they often have a positive attitude towards it. This is interesting within the context of hope despite their very poor prognosis. The circumstances of the choice of chemotherapy near the end of life have been explored in a review [32]. An important aspect that facilitates the patient’s decision towards chemotherapy is explained by the fact that patients have less concerns about adverse effects than for example their well health care providers. Further, the patient-physician communication plays a role, as false optimism about recovery results from avoidance of physicians to pronounce a “death-sentence” as well as from avoidance of patients to hear it [33]. However, the reviewers underline that it is still unknown if patients would integrate honest information into their decision making.

The fact that relatives reflect less (we found a lower amount of data for the corresponding categories) shows that they have a different approach by acting in a more pragmatic way. They focus on the present even more than on the past.

### Strengths and weaknesses

As patients with advanced lung cancer and their relatives experience existential burden after diagnosis and during palliative treatment, it is essential to involve them early in evaluation of treatment in order to sustain their individual quality of life. Consequently, exploring their reflection and strategies to handle cancer was an important step to gain an overarching comprehension of their individual situation within the existing health care structure. The study was conducted by a multi-professional team of researchers and clinicians providing for a broad perspective during design and analysis stages. Though, the findings must be interpreted with caution as one limitation of the study was that interviewers varied. Some aspects within the interviews could be variable despite the semi-structured interview guide used.

## Conclusion

Understanding the individual context of patients and their relatives dealing with advanced cancer is relevant not only for physicians, but also for all health care professionals to be able to provide comprehensive care. It is important to address the views and needs of both patients and relatives individually – to understand where they stand in the coping process. It seems that patients struggle even more with the reflection of their illness while relatives try to provide a strong focus on the present trying to balance the changed life situation. This is certainly a challenge, especially in the context of amenability for discussing and planning end-of-life care. There is a need for a better understanding of the patient’s and the relative’s individual situation and thus for improvement in adequate communication opportunities.

Physicians should be sensitized that patients often have unrealistic expectations in palliative tumor specific therapy and that this may be encouraged by their relatives who try to provide a positive attitude as far as possible. Additional psycho-social care interventions should be afforded systematically as shock, disbelief and denial may aggravate the coping process and hinder end-of-life discussions. Future research should be directed to investigate if relatives are able to facilitate patients’ amenability towards end of life care within the palliative care setting.

## Additional file

Additional file 1: Guideline for interviews with patients and relatives. (DOC 63 kb)
