# Supplementary material for: Reflection of illness and strategies for handling advanced lung cancer – a qualitative analysis in patients and their relatives
Source: BMC Health Serv Res. 2017 Mar 2;17:173. doi: 10.1186/s12913-017-2110-x (PMC5333386; doi:10.1186/s12913-017-2110-x)
Supplement: Additional file 1: — Guideline for interviews with patients and relatives. (DOC 63 kb) [file 12913_2017_2110_MOESM1_ESM.doc]

‚Aims of health care and individual quality of life for patients with limited prognoses’

**Guideline for interviews with patients and relatives**

| **General questions:**   1. What are aspects that are distressing in particular at different times of exploration (for patients / for relatives)? 2. Which actions, procedures, surrounding circumstances are helpful and supportive at different times of exploration? 3. Which actions, procedures, surrounding circumstances are complicating at different times of exploration? 4. What kind of individual needs and requirements do patients have towards therapy and towards there limited lifetime? |
| --- |

1. Preparation

- Introduction of interviewer and department
- Reference of independence, taping (audio), privacy protection: (pseudonymisated analysis, no personal data of participants to be circulated, audio recordings will not be circulated to people outside of researchers’ group. Third parties will not be able to have insight into original documentation. After the studies’ end, audio recordings will be deleted irrevocably. (Regulations of the German Data Protection Law are met)
- Voluntariness, signature of written consent paper (if not yet finished), possibility to stop the interview at every moment. Explanation of the single interview’s course.

1. Topic / Introduction / History 10 min

**Please introduce yourself briefly**

- Name and course of illness

1. Experience and Support 45 min

**Please, tell us about your experiences for example concerning**

- physical symptoms
  - Pain
  - Dyspnoe
  - Fatigue
  - Depression
- Health services
  - Life at home
  - Health services at home
  - Caregiver/Relatives
- Family
  - Burden for relatives
  - Changes in role allocation
  - Necessity of care / involvement of children?
  - Experiences with other family members’ illnesses?
- Social aspects
  - Isolation due to ilness?
  - Changes in role allocation
  - Withdrawal of friends?
  - Financial problems?
- Psychological aspects
  - Experience of fragility within a short period of time
  - Handling of finiteness
  - Handling of hope
  - Handling of uncertainty

**Please tell us, which actions or procedures were helpful within the respective situation?**

**Please reflect, what are supportive services that could help to develop/improve health care at the Thorax-Klinik Heidelberg?**

1. Wrap-up 5 min

- Important aspects not yet addressed?
- Perspective of the course of the study and data analysis.
- Interested in results of the study?
- Request to complete the sheet of socio-demographic information (offering support were needed).
- Thanks for participation and appreciation for gaining important insight.
